# Cytokine and Chemokine Responses in Invasive Aspergillosis Following Hematopoietic Stem Cell Transplantation: Past Evidence for Future Therapy of Aspergillosis

**DOI:** 10.3390/jof7090753

**Published:** 2021-09-13

**Authors:** Patcharin Thammasit, Jirapas Sripetchwandee, Joshua D. Nosanchuk, Siriporn C. Chattipakorn, Nipon Chattipakorn, Sirida Youngchim

**Affiliations:** 1 Department of Microbiology, Faculty of Medicine, Chiang Mai University, Chiang Mai 50200, Thailand; patcharin.th@cmu.ac.th; 2 Neurophysiology Unit, Cardiac Electrophysiology Research and Training Center, Faculty of Medicine, Chiang Mai University, Chiang Mai 50200, Thailand; siriporn.c@cmu.ac.th (S.C.C.); nchattip@gmail.com (N.C.); 3 Cardiac Electrophysiology Unit, Department of Physiology, Faculty of Medicine, Chiang Mai University, Chiang Mai 50200, Thailand; 4 Center of Excellence in Cardiac Electrophysiology Research, Chiang Mai University, Chiang Mai 50200, Thailand; 5 Department of Medicine (Infectious Diseases), Albert Einstein College of Medicine, Bronx, NY 10461, USA; josh.nosanchuk@einsteinmed.org

**Keywords:** cytokines, chemokines, *Aspergillus*, invasive pulmonary aspergillosis, hematopoietic stem cell transplantation

## Abstract

Invasive pulmonary aspergillosis is a frequent complication in immunocompromised individuals, and it continues to be an important cause of mortality in patients undergoing hematopoietic stem cell transplantation. In addition to antifungal therapy used for mycoses, immune-modulatory molecules such as cytokines and chemokines can modify the host immune response and exhibit a promising form of antimicrobial therapeutics to combat invasive fungal diseases. Cytokine and chemokine profiles may also be applied as biomarkers during fungal infections and clinical research has demonstrated different activation patterns of cytokines in invasive mycoses such as aspergillosis. In this review, we summarize different aspects of cytokines that have been described to date and provide possible future directions in research on invasive pulmonary aspergillosis following hematopoietic stem cell transplantation. These findings suggest that cytokines and chemokines may serve as useful biomarkers to improve diagnosis and monitoring of infection.

## 1. Introduction

Fungi are among the most extensively distributed microorganisms and are ubiquitous in the environment. However, a small percentage of these remarkable eukaryotes are also major human pathogens. The frequency of opportunistic fungal infections continues to increase due to the expansion in the numbers of immunocompromised hosts [1]. *Aspergillus* species (spp.) are one of the most common medically important opportunistic fungi [2]. Invasive infections with *Aspergillus* spp. are typically considered life-threatening and most frequently occur in immunocompromised individuals such as those receiving chemotherapy, undergoing solid organ transplantation (SOT), or hematopoietic stem cell transplantation (HSCT) [3,4]. Among the human pathogenic species of the genus *Aspergillus*, *Aspergillus fumigatus* is the most common causative agent, followed by *A. flavus*, *A. terreus*, and *A. niger* [5]. In compromised hosts, *Aspergillus* infections most commonly manifest as invasive pulmonary aspergillosis (IPA). The number of patients undergoing transplantation has grown exponentially in recent years, particularly in patients undergoing HSCT for the treatment of hematological malignancy [6]. IPA occurs in 3.6 to 10.3% of allogeneic HSCT recipients leading to a mortality rate of 50 to 80% [6,7].

Aspergillosis is initiated by inhaling *Aspergillus* spores. The early immune response against *Aspergillus*-associated pulmonary disease is mediated by the interaction between inhaled spores (conidia) and immune effector cells [8]. Effective mechanisms of fungal clearance are regulated by the activation of inflammatory programs (e.g., NF-κB pathway and the NLRP3 inflammasome) resulting in a more robust range of pro-inflammatory cytokine and chemokine secretion by epithelial cells, inflammatory monocytes, dendritic cells (DCs), and alveolar macrophages [9]. The recognition and removal of the fungal spores occurs through (i) physical barriers and mechanical defenses of the respiratory tract, (ii) phagocytic processes, (iii) the activity of antimicrobial peptides (AMPs), and (iv) effector cell activation through engagement of pattern recognition receptors (PRRs), which include soluble receptors and cell-bound receptors [8]. The Toll-like receptor (TLR) family, one of the PRRs directed against conserved molecules in pathogens, plays a major role in the recognition of *Aspergillus*. These aspergillosis-related TLRs include TLR-2, TLR-4, and TLR-9 [10,11]. These TLRs potentially induce the production of pro-inflammatory cytokines and reactive oxygen species (ROS) through a MyD88-NF-κB-dependent signaling pathway [12]. In addition to these MyD88-dependent processes in macrophage-mediated responses, the uptake of *A. fumigatus* spores by respiratory epithelia plays a crucial role in either fungal killing the fungus or containing the organisms within the airway epithelial cells’ (AECs) phagosome [13]. The pulmonary innate immune defense by AECs against *A. fumigatus* induces IL-8 secretion and is controlled by MyD88-independent kinase pathways, including PI3K, p38 MAPK, and ERK1/2 [14]. Thus, MyD88-dependent and -independent pathways are engaged in the early biological activity of *Aspergillus* infection and, thus, are potential therapeutic targets for disease modification.

Moreover, the protective capacity of CD4+ T cells is predominantly mediated by the secretion of a complex cytokine milieu that orchestrates a defensive response [15]. However, dysregulated production of T-helper (Th) cell cytokines is also implicated in the pathogenesis of invasive aspergillosis (IA) [8]. The interplay between environmental conditions, fungal virulence factors, and the host immune response plays a crucial role in the pathogenesis of IA through a variety of mechanisms [9]. Furthermore, cytokine production by CD4+ T helper cells is required for the initiation of the innate response in the IA mouse model [16], as well as in the serum of patients suffering from IPA, compared to healthy individuals [17,18]. Since cytokines act as important mediators of an effective immune response and the regulators for the innate and adaptive immune system [19], the dynamic of cytokines appears to play an important role in the host immune response against *Aspergillus*. Relatively immunocompromised hosts, such as patients undergoing SOT or HSCT, present host conditions that contribute to the overall pathogenic potential of *Aspergillus* infection. HSCT, in particular, is known as a primary immune deficiency exacerbated by the administration of immunosuppressive drugs, resulting in significant CD8 regulatory T cell (Tregs) dysfunction and phagocytic cell defects [20]. Therefore, the complex cytokine and chemokine behaviors produced in response to IA have provided crucial insights into the pathogenesis of IA, and the tracking of changes in these molecules may potentially help guide clinicians in decisions regarding the prophylaxis for and treatment of aspergillosis, especially in HSCT patients.

## 2. Cytokines and Chemokines Responses in Invasive Aspergillosis after Hematopoietic Stem Cell Transplantation

Cytokines and chemokines are biologically active secreted proteins released by immune cells that play critical roles in cell-to-cell communication. In aspergillosis, they are an important component of host defenses against infection by promoting the initiation, maintenance, and resolution of the host response [21]. Innate immune cells composed of granulocytes, monocytes, AECs, and DCs are the first line of defense against *Aspergillus* and are the cells that primarily combat the fungus within the first week after infection [21]. In addition, macrophages phagocytose *Aspergillus* conidia and inhibit their intracellular germination in the early phase of infection [22], which induces the expression of inflammatory chemokines and cytokines. Furthermore, neutrophils and circulating monocytes damage hyphae by secreting oxidative and non-oxidative microbicidal compounds [23]. Hence, early neutropenia followed by immunosuppressive drugs in HSCT leads to defects in certain immune-related phagocytosis. Thus, these findings indicate that the association between HSCT and the immune system is highly dynamic [24].

The results from in vitro analyses reveal that infection of the immature dendritic cells (iDCs) with small germinating conidia (approximate size, 3–8 µm) significantly increased the secretion of specific cytokines (IL-6, IL-12, TNF-α, and IL-10) and chemokines (IL-8, CCL20, and CXCL10) and the expression of immune receptors (PTX3, CXCR4, CCRL2, and IL2RA) [25]. The significant increase of both the pro-inflammatory cytokine TNF-α and chemoattraction chemokines IL-8, CCL-20, and CXCL10 were also observed with stimulation by the *Aspergillus* antigen 18-kDa RNase Aspf1 [26], compared to the levels expressed by unstimulated DCs.

Natural killer (NK) cells are lymphoid cells in peripheral blood that play a critical role in the innate host defense and their cell numbers are related to the severity of IPA [24]. NK cells are known for their release of cytokines and play a unique role in the early phase of an immune response against *Aspergillus* [27]. In vitro infection of human NK cells by *A. fumigatus* hyphae for 6 h increases the secretion of inflammatory cytokines, such as IFN-γ, TNF-α, and growth factor GM-CSF, as well as several chemokines, including CXCL8/IL-8, CCL3/MIP-1α, CCL4/MIP-1β, and XCL1/lymphotactin [27]. Supporting the results from in vitro studies, a murine intranasal infection model using *A. fumigatus* conidia suggested that susceptibility to IA is associated with the levels of genes encoding IL-5 (a Th2 cytokine involved in B cell and eosinophil activation) and IL-17a (a Th17 inflammatory cytokine produced by T cells and NK cells). The increased expressions of the genes encoding IFN-γ, high levels of TNF-α and the upregulation of a network of TNF-α–related genes were significantly related to *Aspergillus* infection [28]. Additionally, the expression of classical Th2 cytokines (IL-4, IL-5, IL-13) was found in bronchiole epithelial lung homogenates of *Aspergillus* protease-induced murine inhalation model compared to the PBS-treated controls [29].

Several studies have demonstrated an alteration of cytokines and chemokines in patients with hematological malignancy undergoing HSCT who subsequently develop invasive fungal disease [18,30,31] and IA in particular [18,32,33]. For example, in adult hematology patients with proven/probable invasive fungal disease (IFD), increases of serum cytokine levels of IL-15 and IL-2R as well as chemokines levels of CCL2 and MIP-1α were observed, whereas the level of IL-4 was significantly lowered, compared to those with no evidence of IFD [18]. Another study in adult hematology patients with probable/possible IA reported higher levels of cytokine IL-6 and chemokine IL-8 in serum and significant elevations in bronchoalveolar lavage (BAL) fluid levels of IL-8, compared to those with other infections [32]. In support of these findings, Gonçalves et al. demonstrated that the BAL fluid levels of cytokines IL-1β, IL-6, IL-17A, IL-23, TNF-α, and chemokine IL-8 were increased in patients diagnosed with IA, which were also consistent with levels of these cytokines in serum [33]. Notably, although the expression of in vitro and in vivo cytokines/chemokines varies in the different studies, these discrepancies may be explained by differences in cell types responding to *Aspergillus* stimuli and the different patient populations. However, all these laboratory findings suggest that the elevation of cytokines/chemokines in serum and BAL fluid levels were associated with increased risk of IA and, thus, may be used as a valuable indicator of the risks associated with development of IA and guide enhanced antifungal prophylaxis and early treatment. These findings are summarized in Table 1.

## 3. Genetic Polymorphisms in Hematopoietic Stem Cell Transplantation Patients Associated with Invasive Aspergillosis

Given the variable risk of infection and its clinical outcome among patients with comparable predisposing factors, genetic predisposition is considered as the most important factor of individual susceptibility to IA [34]. *Aspergillus* conidia or hyphae interact with the innate immune system through PRRs, which included Dectin-1, TLR-2, and TLR-4 [35]. These TLRs can trigger PI3K, MAPK, and ERK1/2 signaling pathways, resulting in the production of several cytokines and chemokines including IL-8, IL-1α, IL-1β, IL-17, TNF-α, CCL3, CCL4, as well as CXCL1 from immune cells [36,37]. Dectin-1 is an NK-cell-receptor-like C-type lectin that is widely expressed on monocytes, macrophages, DCs, neutrophils, and eosinophils [38]. Dectin-1 mediates antifungal immunity through the promotion of inflammatory activity, eventually leading to fungal clearance. Antifungal immunity can occur through the triggering of Syk, which leads to the induction of NF-κB and production of protective cytokine responses. Dectin-1 signals and induces the production of cytokines through a Syk-independent pathway (noncanonical NF-κB pathway) [39]. Therefore, genetic polymorphisms affecting human Dectin-1 can be partly attributed to defective cytokine production, leading to an increased susceptibility to IA. Defective production of TNF-α and IL-6 has been found in both PBMCs and BEAS-2B respiratory epithelial cells harboring the Dectin-1 Y238X polymorphism [40,41]. Additionally, Dectin-1 knockout in BALB/c mice have decreased production of IFN-γ, IL-17A, and IL-10, and have a significantly reduced ability to control *Aspergillus* infection [41]. Conversely, single nucleotide polymorphisms (SNPs) in the intracellular PRR NOD2 can decrease the risk of IA [35]. NOD2 deficiency results in a defective inflammatory response with alterations in the levels of IL-1β, IL-17A, IL-22, and IFN-γ produced by PBMCs from hematological patients undergoing allogeneic HSCT, and IL-6 and TNF levels in Nod2^-/-^ deficient mice [42]. Furthermore, low levels of serum IL-10 and IL-8 have been reported in patients with hematological malignancies undergoing allogeneic HSCT [42]. Thus, targeting assays for alternations in NOD2 may be an attractive method in personalized management strategies for IA. However, at present, these findings fundamentally show that defects in NOD2 potentially reduce *Aspergillus*-induced cytokine driven inflammation. Importantly, it needs to be elucidated whether cytokine alterations mechanistically protect from fungal infection in HSCT patients with NOD2 variants. Furthermore, polymorphisms in other cytokine genes such as IL-1 and IL-10 have also been implicated as genetic biomarkers of susceptibility to IFD [43,44]. These findings are summarized in Table 2.

## 4. Drug-Related Cytokine Alterations in Developing Invasive Aspergillosis in Hematopoietic Stem Cell Transplantation Patients

Diverse medications are administered to patients undergoing HSCT, and many of these are immunomodulatory and impair inflammatory responses. For example, the TNF-α receptor blocker etanercept has emerged as a useful therapeutic for chronic and acute graft-versus-host disease (GvHD) management, and it also has a significant influence on the immune response against *Aspergillus* [46]. Etanercept fuses to the TNF receptor 2 to block the release of this key pro-inflammatory cytokine, and the reduction of TNF-α limits its downstream signaling including impairing NF-κB activation [47]. A recent in vitro study using monocyte-derived macrophages (MDM) stimulated with *A. fumigatus* showed a significant reduction of chemokine CXCL10 release after etanercept application [46]. Consistent with the in vitro results, patients receiving etanercept with probable IA have reduced CXCL10 serum concentrations [46]. Hence, etanercept administration markedly reduces CXCL10 level, which is associated with a decreased potency of host defensive mechanisms against *Aspergillus*. These effects may be partly explained by the important role of TNF-α in activating CXCL10 secretion via the STAT1-NF-κB1(p50)-RelA(p65) pathway [48]. CXCL10 exhibits a strong chemotactic property for immune cells, which is important for defense against fungal infection [49].

Cyclosporine is another medication frequently administered in the setting of HSCT. Cyclosporine treatment in an anti-*Aspergillus* T_H_1 model using cells isolated from normal human volunteers revealed that this immunosuppressant significantly reduced the number of IFN-γ producing cells and suppressed the levels of the cytokine. Furthermore, cyclosporine significantly decreased the expression of CD154 and increased apoptosis rate on anti-*Aspergillus* T_H_1 cells [50]. These modifications induced by cyclosporine increase the risk for IA.

Recombinant human granulocyte colony stimulating factor (r-metHuG-CSF, or GCSF) has been widely used to mitigate radiation-induced oropharyngeal mucositis. In contrast to etanercept and cyclosporine, GCSF enhances the activities of neutrophils against *Aspergillus* infection by increasing the number of mature neutrophils, enhancing phagocyte oxidative responses and increasing phagocytic activity [51]. These responses are associated with a benefit for the prevention and treatment of IA in transplant patients who display an impaired respiratory burst [52]. In addition, a recent in vivo study demonstrated that macrophage colony-stimulating factor (M-CSF) also has a therapeutic benefit against *Aspergillus* in HSCT and progenitor cells-transplanted mice by inducing the differentiation of myeloid progenitor cells [53]. Thus, administration of M-CSF not only reduced graft-versus-host disease in HSCT [54], but it can augment protective effector responses to *Aspergillus* after HSCT in patients.

In summary, the drugs administered to HSCT patients may significantly influence their risk for IA, and drug regimens should be considered for the personalized risk stratification protocols in these patients. These findings are summarized in Table 3.

## 5. Pattern of Cytokine Production in Dendritic Cells (DCs) Activation for Vaccine Prospects against Invasive Aspergillosis in Hematopoietic Stem Cell Transplantation

Cell-mediated immunity (CMI) plays a vital role in protection against *Aspergillus* [55]. The cellular response of the airway bronchial epithelial cells to *Aspergillus* is the first layer of protection against the pathogen. The major roles of bronchial epithelial cells include enhancing recruitment of macrophages and neutrophils, increasing the release of pro-inflammatory cytokines and chemokines, and activating and skewing T-cell subsets [56]. The engulfment of conidia and/or hyphae by alveolar macrophages leads to the release of numerous cytokines and chemokines that further enhance the recruitment of innate effector cells including macrophages and DCs [57]. DCs act as the key player in connecting innate and adaptive immune response against *Aspergillus* by specifically activating naive T-cells and their differentiation into an effector lineage [58].

Several reports from in vitro studies have demonstrated that murine and human DCs are activated by *A. fumigatus* morphotypes [59,60,61,62]. Murine DCs increased their production of IL-10 and IL-12 in response to *Aspergillus* conidia, and hyphae stimulated the production of both IL-4 and IL-10. Human DCs are also responsible for the initiation of both the innate and the adaptive immune response [59,60,61,62]. The differences in cytokine production patterns for conidia and hyphae might be explained by the variable functional activation of DCs in response to the different morphotypes of *A. fumigatus*. For example, these cells may internalize conidia or hyphae of *Aspergillus* via different phagocytic mechanisms, and engage PRRs [63]. In addition, DCs also play an important role in Th17 polarization during *A. fumigatus* infection [64]. Activation of IL-17 cytokine leads to the recruitment of neutrophils and increased concentrations of defensins that ultimately cause inflammation. A heightened IL-23/IL-17-dependent inflammatory response is also associated with susceptibility to aspergillosis in the mice model, and modulation of these cytokines is essential in the early control of the infection [65,66]. Consequently, these differences in interactions of DCs can lead to divergent patterns of cytokine production. DC vaccines for fungal diseases are an exciting therapeutic approach to protect compromised patients [67]. The power of DC vaccines is demonstrated by the striking capacity of primed DCs to protect HSCT mice. Perruccio et al. demonstrated that the infusion of murine splenic DCs infected with *A. fumigatus* conidia decreased lung CFUs and increased the median survival time of HSCT mice with IA [59]. DC vaccination also increased the levels of IFN-γ and IL-10, and demonstrated a Th1-protective response [59]. Others have reported that murine splenic DCs infected with *A.* *fumigatus* produced increased amounts of IFN-γ and IL-4 after internalization of conidia [61]. Murine splenic DCs only increase IL-4 after internalization of hyphae [61]. Interestingly, adoptive transfer in vivo of purified DCs pulsed with conidia or hyphae resulted in priming of CD4+ T cells for Th1 cytokine (IFN-γ and TNF-α) or Th2 cytokine (IL-4 and IL-10) production, respectively, which is similar to what was described above for in vitro DC responses to these morphotypes. Hence, these results underscore the major role of DCs in the polarization of T cells and patterns of susceptibility or resistance during IA [68]. These findings also reinforce that morphogenesis is a key fungal virulence factor as the transition from conidia to hyphae induces different host responses and the host must alter immunological tactics in order to control disease.

These studies confirm that DCs are important for host response against *Aspergillus* and suggest that the functional plasticity of DCs in response to *Aspergillus* infection can be potentially therapeutically exploited. *Ex vivo*-generated and primed DCs, in particular, might be useful for restoring pathways of cell-mediated immunity or enhancing antifungal immunity following HSCT. These findings are summarized in Table 4.

## 6. Conclusions and Perspectives

**Figure 1:**
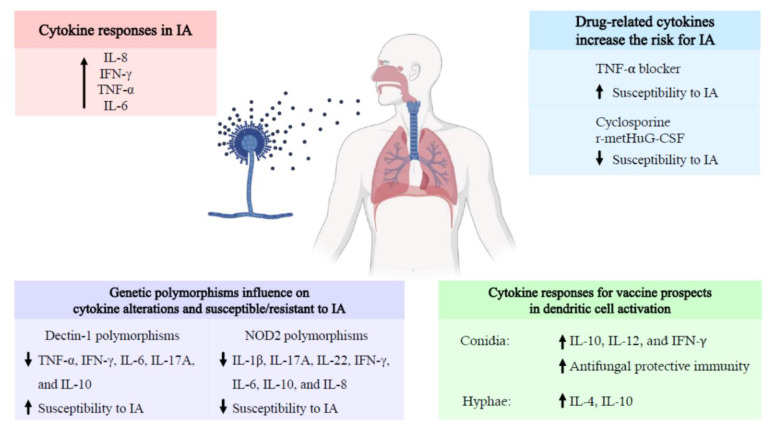
Schematic representation of the cytokine responses associated with invasive aspergillosis in hematopoietic stem cell transplantation. Cytokine responses related to invasive aspergillosis associated with an increase the risk of the disease. Both drugs and genetic polymorphism influence the cytokine expression profile during hematopoietic stem cell transplantation. In addition, different activation patterns of *Aspergillus* morphotypes lead to different cytokine expression profiles. Abbreviations: IA, invasive aspergillosis; IFN, interferon; IL, interleukin; NOD2, nucleotide oligomerization domain 2; r-metHuG-CSF, recombinant human granulocyte colony stimulating factor; TNF, tumor necrosis factor. The black arrows indicate the increase or decrease in cytokines and chemokines.

A correlation of certain infectious diseases with an alteration of cytokine profiles has been deemed clinically useful for disease management, with COVID-19 being the most recent example where modifying specific cytokines can effectively mitigate disease [69,70]. One of the major roles of cytokines in the immune system, as immune regulators, are their pleiotropic effects, which include immunologic, hematopoietic, and pro-inflammatory activities. IA is correlated with remarkable cytokine alterations, as reflected by the dynamic changes in cytokine and chemokine levels. Alterations in immunity, particularly defects in innate immunity, is one of the concerning factors following HSCT and this immune compromise significantly increases risk for IA. The findings in this review, especially the data on IL-8 and pro-inflammatory cytokines (IFN-γ, TNF-α, and IL-6), could therefore be considered a biological characteristic that may serve as a platform for, primarily, analysis of patients targeted for IA monitoring. Additionally, these molecules and associate pathways are attractive potential therapeutic targets for modifying the pathogenesis of IA. Nevertheless, other confounding factors such as drugs being administered to patients and genetic polymorphisms in patients should be considered, as these will alter biological response patterns (see Figure 1). Hence, there is an urgent need to better understand the different contributions of the various factors—host and therapeutics—that underly the cytokine signaling pathways in these patients with and without IA. Furthermore, it is important to consider that other invasive fungal infections, and some bacterial pathogens, may trigger host immune responses with similar immune response profiles. Thus, the assumption that there is a causal association between particular cytokine/chemokine profiles and the specific occurrence, development, and resolution of IA requires further investigation. Additionally, research is required to identify the cut-off levels of the key cytokines for their use as diagnostic tools in HSCT patients with IA. This review highlights that targeting cytokine alterations is a promising method for predicting the risk for, and progression of, IA in HSCT patients, and this approach may also be used to monitor the efficacy of antifungal prophylaxis and therapy.

## Figures and Tables

**Table 1:** Cytokine and chemokine responses in invasive aspergillosis after hematopoietic stem cell transplantation.

Models	Samples	Methods	Major Findings			Interpretation	Ref.
			Cytokines	Chemokines	Others		
In vitro							
iDC + *A. fumigatus*-small germinating conidia (6 h of stimulation)	Infected iDCs	qRT-PCR	↑ IL-6 ↑ IL-12 ↑ TNF-α ↑ IL-10	↑ IL-8 ↑ CCL20 ↑ CXCL10	↑ PTX3 ↑ TLR-2 ↓ TLR-4	*A. fumigatus* germ tubes induced the expression of genes associated with recognition and phagocytosis in iDCs with a time-dependent manner.	[25]
iDC *+* *A. fumigatus* antigen Aspf1	Infected iDCs	qRT-PCR	↑ TNF-α	↑ L-8 ↑ CXCL10 ↑ CCL20	−	Aspf1, a member of a family of conserved RNases, induces a pro-inflammatory cytokine response.	[26]
NK cells obtained from PBMCs + *A. fumigatus* hyphae (6 h of stimulation)	Infected NK cells	qRT-PCR	↑ IFN-γ ↑ TNF-α ↑ GM-CSF	↑ CXCL8 /IL-8 ↑ CCL3/MIP-1α ↑ CCL4/MIP-1β ↑ XCL1/ lymphotactin	↓ NKp30 ↑ CD56	NK cells reveal the expression and release of immunomodulatory molecules involved in antifungal immune responses.	[27]
**In vivo**							
Mice CD1 strain infected by intranasal instillation with *A. fumigatus* conidia (*N* = 24)	Mouse whole-lung homogenates	qRT-PCR ELISA	Immunpetent mice: Infted vs. Saline controls ↑ IL-17a mRNA ↑ TNF-α protein level			Susceptibility to IA is associated with a high level of TNF-α at the site of infection and the upregulation of a network of TNF-α–related genes.	[28]
			Immunosuppressed mice: Infected *vs.* Saline controls ↑ IFN-γ mRNA ↑ IL-17a mRNA ↑ TNF-α protein level ↑ IL-5 protein level				
			Immunocompetent *vs.* Immunosuppressed mice ↓ TNF-α ↓ IFN-γ ↓ IL-4 ↓ IL-12p40				
BALB/c mice infected by intranasal instillation with *A. fumigatus* proteases, *Asp f 5* and *Asp f 13* (*N* = 20)	Mouse lung homogenates	ELISA	Infected *vs.* PBS controls ↑ IL-4 ↑ Serum IgE ↑ IL-5 ↑ IL-13			*A. fumigatus* secreted allergen proteases, *Asp f 5* and *Asp f 13*, are important for induction of Th2 cytokines secretion and increased IgE levels, which are fundamental features of allergic asthma and an indication of disease severity.	[29]
**Clinical study**							
Adult hematology patients with proven/probable IFD (*N* = 172)	Serum	ELISA	↑ IL-15 ↑ IL-2R ↓ IL-4	↑ CCL2 ↑ MIP-1α	−	High IL-2R and CCL2 concentrations as indicators for the risk of developing IFD.	[18]
Adult hematology patients with probable/possible IA (*N* = 43)	BAL Serum	ELISA	Serum ↑ IL-6	BAL ↑ IL-8 Serum ↑ IL-8	↑ *Aspergillus*-specific lateral-flow device test	High serum IL-8 levels were highly specific and highly sensitive for the diagnosis of IA.	[32]
Patients diagnosed with IA (*N* = 48)	BAL Serum	ELISA	BAL ↑ IL-1β ↑ IL-6 ↑ IL-17A ↑ IL-23 ↑ TNF-α Serum ↑ IL-6 ↑ IL-17A ↑ IL-23	BAL ↑ IL-8 Serum ↑ IL-8	↑ Galactomannan in BAL specimens	Alveolar cytokines might be useful in supporting current diagnostic approaches for IPA biomarkers. IL-8 was the best performing analyte with the most relevant discriminator between cases of IPA and controls.	[33]

Abbreviations: BAL, bronchoalveolar lavage; CCL, chemokine (C-C motif) ligand; CD, cluster of differentiation; CXCL, chemokine (CXC motif) ligand; ELISA, enzyme-linked immunosorbent assay; GM-CSF, granulocyte-macrophage colony-stimulating factor; h, hour; IA, invasive aspergillosis; iDCs, immature dendritic cells; IFD, invasive fungal disease; IFN, interferon; IgE, immunoglobulin E; IL, interleukin; IPA, invasive pulmonary aspergillosis; MIP, macrophage inflammatory proteins; mRNA, messenger RNA; NK cell, natural killer cell; PBMCs, peripheral blood mononuclear cells; PBS, phosphate buffered saline; PTX, paclitaxel; qRT-PCR, quantitative reverse transcription polymerase chain reaction; TLR, toll-like receptor; TNF, tumor necrosis factor. The black arrows indicate the increase or decrease in cytokines and chemokines.

**Table 2:** Genetic polymorphisms in hematopoietic stem cell transplantation patients are associated with susceptibility/resistance to invasive aspergillosis.

Models	Polymorphism	Major Findings		Interpretation	Ref.
		Cytokines	Others		
In vitro					
PBMCs	Dectin-1 Y238X Stop Codon Polymorphism + heat-killed *A. fumigatus* hyphae + live *A. fumigatus* conidia	↓ TNF-α ↓ IL-6	↓ binding ability to β-glucan	Dectin-1 Y238X resulted in the reduction of pro-inflammatory cytokines due to the Dectin-1 receptor, which is known to play a role in fungal cell wall β-glucan recognition.	[40]
BEAS-2B (Respiratory epithelial cells)	Dectin-1 blockade by siRNA + Stimuli (β-glucan or *Aspergillus* conidia)	↓ IL-6 ↓ TNF-α	−	Dectin-1 expressed on epithelial cells contributes to the production of cytokines.	[41]
PBMCs from allogeneic HSCT	*NOD2* genetic variation - P268S (TT-genotype) + *A. fumigatus* conidia - complete NOD deficiency + *A. fumigatus* conidia	Infected in TT-genotype compared with infected in CC-and CT-genotype ↓ IL-1β ↓ IL-17A *Aspergillus* infected compared with uninfected ↓ IL-1β ↓ IL-22 ↓ IFN-γ	*Aspergillus* infected compared with uninfected ↓ IL-17A^+^, IL-22^+^, and IFN-γ^+^ CD4 T-cell populations	Human *NOD2* deficiency reduces *Aspergillus*-induced inflammatory cytokines.	[42]
Human PMBCs from solid-organ transplant recipients	*IL1B rs16944* SNP + *A. fumigatus* conidia *IL1RN rs419598* SNP + *A. fumigatus* conidia	IL1B rs16944 SNP ↓ IL-1β ↓ TNF-α ↓ IL-22 IL1RN rs419598 SNP ↓ IL-1β ↓ TNF-α	−	Both IL1B rs16944 and IL1RN rs419598 SNPs effect *Aspergillus*-induced cytokine release.	[43]
Macrophages from healthy blood donors	*IL10* SNP with GG genotype + *A. fumigatus* conidia	↓ IL-10 ↓ TNF-α ↓ IL-6 ↓ IL-1β ↓ IL-8	↓ fungal clearance	IL-10 overexpression influences IA by suppressing antifungal immunity.	[44]
**In vivo**					
BALB/c mice with HSCT + *Aspergillus* (*N* = 16)	Dectin-1 knockout mice	↓ IFN-γ ↓ IL-17A ↓ IL-10	↑ fungal growth	Dectin-1 modulates immunity and tolerance *via* IFN-γ / IL-10 production, and both cytokines activate the protection of Th1/Treg antifungal responses.	[41]
*Nod2*-deficient (*Nod2*^-/-^) C57BL/6 mice + *Aspergillus* (lethal dose) (*N* = 22)	*Nod2*^-/-^ deficient mice (Splenocytes)	↓ IL-6 ↓ TNF	↑ 14-day survival	NOD2 augments *Aspergillus*-induced cytokine responses and results in resistance to *Aspergillus* infection.	[42]
**Clinical study**					
Patients who developed IA post HSCT (*N* = 71) Non-HSCT patients with IA (*N* = 21)	Y238X Stop Codon Polymorphism	−	↑ susceptibility to IA	Dectin-1 Y238X heterozygosity had a limited influence on susceptibility to IA.	[45]
Hematological patients undergoing allogeneic HSCT (*N* = 310)	*NOD2* genetic variation - P268S SNP	↓ serum IL-10 ↓ serum IL-8	↓ susceptibility to IA	Genetic deficiency of *NOD2* results in an alteration of cytokine production in response to *Aspergillus* infection.	[42]
An allograft with IA (*N* = 81) or without IA (*N* = 58)	*CXCL10* genetic variation - C+11101T - C+1642G - A1101G	↑ serum CXCL-10	↑ susceptibility to IA	Polymorphisms in CXCL10 altered chemokine secretion and increased the risk of IA after alloSCT.	[40]

Abbreviations: alloSCT, allogeneic stem cell transplantation; CXCL, chemokine (CXC motif) ligand; HSCT, hematopoietic stem cell transplant; IA, invasive aspergillosis; IFN, interferon; IL, interleukin; NOD, nucleotide oligomerization domain; PBMCs, peripheral blood mononuclear cells; SNP, single nucleotide polymorphism; Th cell, T helper cell; TNF, tumor necrosis factor. The black arrows indicate the increase or decrease in cytokines and chemokines. *Nod2*^-/-^ indicates complete deletion (-) of *Nod2* alleles.

**Table 3:** Drug-related cytokine alterations associated with invasive aspergillosis in hematopoietic stem cell transplantation patients.

Models	Study Protocol	Study Pethods	Major Findings			Interpretation	Ref.
			Cytokines	Chemokines	Others		
In vitro							
Human MDM	*A. fumigatus* (MOI 0.5) with 2 µg/mL of TNF-α blocker, Etanercept for 6 h	ELISA qRT-PCR	↓ TNF-α	↓ CXCL10	↓ *RELB* ↓ *ICAM1* ↓ *BCL3* ↓ *BIRC3*	Etanercept lowered inflammatory cytokines and chemokines as well as downregulated genes involved in TNF-α signaling, which offers new data regarding risk factors for IA and the administration of etanercept.	[46]
PBMCs from healthy volunteers (*N* = 8)	Generation of anti-*Aspergillus* Th1 cells + Cyclosporine	ELISA FACS analysis	↓ IFN- γ	−	↓ anti-*Asper*. Th1 cells ↓ CD154 ↑ apoptosis rate	Cyclosporine suppresses human anti-*Aspergillus* Th1 cells.	[50]
Neutrophils	+ *A. fumigatus* +/− r-metHuG-CSF	FACS analysis	−	−	↑ number of mature neutrophils ↑ respiratory burst ↑ phagocytic activity	G-CSF enhances the activities of neutrophils against *Aspergillus* spp.	[52]
**In vivo**							
Irradiated and HS/PC-transplanted mice (*N* = 13)	10 µg of recombinant M-CSF + *A. fumigatus*	FACS analysis Fungal load	−	−	↑ myeloid differentiation ↓ fungal tissue load	M-CSF has a beneficial effect against severe infections after transplantation.	[53]
**Clinical study**							
Serum samples from patients with probable IA (*N* = 8)	TNF-α blocker treatment	ELISA	↓ CXCL10	−	−	TNF-α blocker reduces CXCL10 serum concentrations in patients with probable IA.	[46]

Abbreviations: CXCL, chemokine (CXC motif) ligand; FACS, fluorescence-activated cell sorting; HSCT, hematopoietic stem cell transplant; h, hour; HS/PC, hematopoietic stem and progenitor cells; IA, invasive aspergillosis; IFN, interferon; IL, interleukin; M-CSF, macrophage colony-stimulating factor; MDM, monocyte-derived macrophages; MOI, multiplicity of infection; PBMCs, peripheral blood mononuclear cells; r-metHuG-CSF, recombinant human granulocyte colony stimulating factor; SNP, single nucleotide polymorphism; T_H_ cell, T helper cell; TNF, tumor necrosis factor. The black arrows indicate the increase or decrease in cytokines and chemokines.

**Table 4:** Perspective of vaccine development using dendritic cells (DCs) activation and its impact on cytokine levels in invasive aspergillosis.

Models	Study protocol	Major Findings in DCs Pulsedwith Fungal Morphotypes					Interpretation	Ref.
		Conidia	Conidial RNA	Hyphae	Hyphal RNA	Others		
In vitro								
Murine DCs	*A. fumigatus* application for 24 h	↑ IL-12 ↑ IL-10	↑ IL-12 ↑ L-10	↑ IL-4 ↑ IL-10	↑ IL-4 ↑ IL-10	↑ MHC class II antigen	**Murine DCs** - mainly produced IL-12 in response to conidia or the corresponding RNA - produced IL-4/IL-10 in response to hyphae or the corresponding RNA.	[59]
Human Myeloid DCs (MDCs)	1. *A. fumigatus* application for 24 h *2. A. fumigatus* + activated cytokines producing CD4^+^ Th cells	Group 1 ↑ IL-12 ↑ IFN-α ↑ IL-10 Group 2 ↑ IFN-γ	Group 2 ↑ IFN-γ	Group 1 ↑ IFN-α ↑ IL-10 Group 2 ↑ IFN-γ ↑ IL-10 ↑ IL-4	Group 2 ↑ IFN-γ ↑ IL-10 ↑ IL-4	−	MDCs mainly produced IL-12 after *Aspergillus* infection. Upon pulsing with conidia, MDCs mainly activated IFN-γ producing CD4^+^ Th1 cells.	[59]
Human plasmacytoid DCs (PDCs)	1. *A. fumigatus* application for 24 h *2. A. fumigatus* + activated cytokines producing CD4^+^ Th cells	Group 1 ↑ IFN-α ↑ IL-10 Group 2 ↑ IFN-γ ↑ IL-10	Group 1 ↑ IL-12 ↑ IFN-α ↑ IL-10 Group 2 ↑ IFN-γ ↑ IL-10	Group 2 ↑ IFN-γ ↑ IL-10 ↑ IL-4	Group 1 ↑ IFN-α ↑ IL-10 Group 2 ↑ IFN-γ ↑ IL-10 ↑ IL-4	−	PDCs produced IL-10 and IFN-α in response to *Aspergillus fumigatus*. Upon pulsing with conidia, PDCs mainly activated IFN-γ- and IL-10-producing CD4^+^ cells.	[59]
Murine lung myeloid DCs	*A. fumigatus* application for 24 h	↑ TNF-α ↑ IL-12 p70	−	↑ TNF-α ↑ IL-4 ↑ IL-10	−	−	Upon exposure to *A. fumigatus* conidia or hyphae, pulmonary DC differentially produce IL-12 and IL-4/IL-10.	[60]
Murine DCs	*A. fumigatus* application for 24 h	↑ IL-12 p70 ↑ IL-10	↑ IL-12 p70	↑ IL-4 ↑ IL-10	↑ IL-4 ↑ IL-10	−	Murine DC produced mainly IL-12 in response to conidia and IL-4 and IL-10 in response to hyphae.	[61]
Human DCs	*A. fumigatus* application for 24 h	↑ IL-12	−	−	−	↑ HLA class II ↑ CD80 ↑ CD86	DCs produced IL-12 in response to *A. fumigatus* conidia.	[62]
Cocultures of autologous DCs with lymphocytes	*A. fumigatus* application for 24 h	↑ IFN-γ	−	−	−	−	*A. fumigatus* stimulation of lymphocytes through autologous DC results in a type-1 polarization (protection against aspergillosis).	
Human DCs	1. recombinant *A. fumigatus* antigens + 18-kDa RNase Aspf1 2. recombinant *A. fumigatus* antigens + putative glycosidase Crf1	−	−	−	−	Group 1 ↑ IL-8 ↑ IL-23 ↑ CXCL10 ↑ CCL20 Group 2 ↑ IL-8 ↑ CXCL10 ↑ CCL20	The interactions between human immature dendritic cells and *A. fumigatus* antigens triggered the increased level of expression of genes encoding pro-inflammatory cytokines and chemokines.	[26]
**Adoptive transferred *Aspergillus*-pulsed DCs in vivo**								
Murine splenic DCs	Pulsed with *Aspergillus* conidia and administered into recipient HSCT mice	−	↑ IFN-γ ↑ IL-10	−	−	↑ Median survival time ↓ CFU in the lungs	Adoptively transferred fungus RNA-transfected dendritic cells induce Th1-mediated resistance to fungal infections in mice with allogeneic HSCT.	[59]
Murine splenic DCs	Pulsed with *Aspergillus* and administered into recipient HSCT mice	↑ IFN-γ ↑ IL-4	↑ IFN-γ ↑ IL-4	↑ IL-4	↑ IL-4	−	Adoptively transferred fungus pulsed dendritic cells induce T_H_ priming to the fungus in vivo.	[61]

Abbreviations: CCL, chemokine (C-C motif) ligand; CCR, C-C chemokine receptor; CFU, colony forming unit; CXCL, chemokine (CXC motif) ligand; DCs, dendritic cells; HLA, human leukocyte antigen; HSCT, hematopoietic stem cell transplant; IFN, interferon; IL, interleukin; MDCs, myeloid dendritic cells; MHC, major histocompatibility complex; PDCs, plasmacytoid dendritic cells; T_H_ cell, T helper cell; TNF, tumor necrosis factor. The black arrows indicate the increase or decrease in cytokines and chemokines.

## Data Availability

Not applicable.
